# Quantitative SPECT (QSPECT) at high count rates with contemporary SPECT/CT systems

**DOI:** 10.1186/s40658-021-00421-3

**Published:** 2021-10-30

**Authors:** Alessandro Desy, Guillaume F. Bouvet, Étienne Croteau, Nancy Lafrenière, Éric E. Turcotte, Philippe Després, Jean-Mathieu Beauregard

**Affiliations:** 1 Department of Medical Imaging, and Research Centre (Oncology Axis), CHU de Québec – Université Laval, 11 côte du Palais, Quebec City, QC G1R 2J6 Canada; 2 Department of Radiology and Nuclear Medicine, and Cancer Research Centre, Université Laval, Quebec City, Canada; 3 CHUS Research Centre, and Sherbrooke Molecular Imaging Centre, CIUSSS de l’Estrie – CHUS, Sherbrooke, Canada; 4 Department of Nuclear Medicine and Radiobiology, Université de Sherbrooke, Sherbrooke, Canada; 5 Department of Radiation Oncology, and Research Centre (Oncology Axis), CHU de Québec – Université Laval, Quebec City, Canada; 6 Department of Physics, Physical Engineering and Optics, and Cancer Research Centre, Université Laval, Quebec City, Canada

**Keywords:** Quantitative SPECT, SPECT/CT, Calibration, Dead time, High count rate

## Abstract

**Background:**

Accurate QSPECT is crucial in dosimetry-based, personalized radiopharmaceutical therapy with ^177^Lu and other radionuclides. We compared the quantitative performance of three NaI(Tl)-crystal SPECT/CT systems equipped with low-energy high-resolution collimators from two vendors (Siemens Symbia T6; GE Discovery 670 and NM/CT 870 DR).

**Methods:**

Using up to 14 GBq of ^99m^Tc in planar mode, we determined the calibration factor and dead-time constant under the assumption that these systems have a paralyzable behaviour. We monitored their response when one or both detectors were activated. QSPECT capability was validated by SPECT/CT imaging of a customized NEMA phantom containing up to 17 GBq of ^99m^Tc. Acquisitions were reconstructed with a third-party ordered subset expectation maximization algorithm.

**Results:**

The Siemens system had a higher calibration factor (100.0 cps/MBq) and a lower dead-time constant (0.49 μs) than those from GE (75.4–87.5 cps/MBq; 1.74 μs). Activities of up to 3.3 vs. 2.3–2.7 GBq, respectively, were quantifiable by QSPECT before the observed count rate plateaued or decreased. When used in single-detector mode, the QSPECT capability of the former system increased to 5.1 GBq, whereas that of the latter two systems remained independent of the detectors activation mode.

**Conclusion:**

Despite similar hardware, SPECT/CT systems’ response can significantly differ at high count rate, which impacts their QSPECT capability in a post-therapeutic setting.

**Supplementary Information:**

The online version contains supplementary material available at 10.1186/s40658-021-00421-3.

## Introduction

Quantitative SPECT (QSPECT) is increasingly used in the clinics with both diagnostic and therapeutic radionuclides. It is of particular utility in dosimetry-driven protocols of personalized radiopharmaceutical therapy, in which the administered activity can be highly variable [1–4]. We have previously shown the importance of dead-time correction in a ^177^Lu post-therapeutic setting [5]. However, using ^177^Lu and ^99m^Tc, we previously observed divergent behaviours between the two detectors of a SPECT/CT system at high count rate, which lead to image artifacts and limitations in QSPECT accuracy [6, 7]. This motivated us to investigate this issue more thoroughly, and also with other systems. Indeed, patients administered a high activity and/or retaining a high fraction thereof can challenge the QSPECT capability of a SPECT/CT system. In a post-therapeutic setting, this can translate into increased dosimetric errors, as well as suboptimal planning and delivery of a personalized radiopharmaceutical therapy.

In this work, we evaluated key quantitative characteristics of three dual-head NaI(Tl)-crystal SPECT/CT systems from two major vendors at high count rate using ^99m^Tc (for practicality), including the per-detector response.

## Materials and methods

### SPECT/CT systems

Three contemporary dual-head Anger systems with 9.5 mm-thick NaI(Tl) crystals and a multislice CT subsystem were evaluated: System A was a Symbia T6 SPECT/CT (Siemens Healthineers, Germany). Systems B and C were, respectively, Discovery NM/CT 670 and NM/CT 870 DR (GE Healthcare, USA). Systems A and B were equipped with low-energy high-resolution collimators, while System C, with low-energy high-resolution and sensitivity ones. All energy depositions in the detector cause dead time [6, 8, 9]. Therefore, along with the ^99m^Tc 140-keV photopeak (20% width) and the lower and upper scatter windows (10%), three additional energy windows were added to cover a wide spectrum (18 to 504 keV). All acquisitions were repeated with only the detector 1, only the detector 2, and both detectors activated. The existence of a “fast” mode on Systems B and C was brought later to our attention and was not activated for any experiment. However, it was confirmed by the vendor that no such mode exists on System A.

### Planar acquisitions

For planar acquisitions, twenty 0.5-mL Eppendorf tubes were filled each with approximately 600 (System A) and 700 MBq (System B and C) of ^99m^Tc. The tubes were placed 3 cm apart on a 30 × 23 cm, 5 mm-thick cardboard positioned equidistantly between the detectors [8]. Dynamic planar acquisitions (40 frames; 15 s/frame; 256 × 256 matrix) were performed, while adding the tubes one at a time on even-numbered frames, so that the activity was constant during odd-numbered ones. The experiment was repeated after 20 h of decay. The total activity thus ranged from 60 MBq up to 12 GBq (System A) and from 70 MBq to 14 GBq (Systems B and C).

### Calibration and pileup

The systems under study were assumed to be paralyzable [7, 10]. For System A, we previously ruled out a cascaded paralyzable–non-paralyzable system, as the non-paralyzable constant was smaller than the paralyzable constant [6, 8, 11]. Sorenson described the paralyzable model of Anger cameras as [10]:

1 $$R_{o} = R_{t} \cdot e^{{ - R_{t} \cdot \tau }}$$

where *τ*, R_o_ and R_t_ correspond to the dead-time constant, the observed count rate and the true count rate, respectively. As detailed previously [6, 8], we define the camera calibration factor (CF) as the dead-time-free primary photons count rate (scatter-subtracted photopeak count rate) per activity (A). Equation (1) can be modified to express the wide-spectrum count rate (R_Wo_) in relation with the observed primary photons count rate (R_Po_) as follows [6, 8], [10]:

2 $$R_{Wo} = CF \cdot A \cdot \frac{{R_{Wo} }}{{R_{Po} }} \cdot e^{{ - CF \cdot A \cdot \frac{{R_{Wo} }}{{R_{Po} }} \cdot \tau }}.$$

CF and *τ* were determined with the full range of quantifiable data obtained during the planar acquisitions by nonlinear regression of Eq. (2) using Python 3.6 (Lmfit package, least-square minimization) [6, 12, 13]. To illustrate the observed vs. expected count rate relationship, we set the expected wide-spectrum count rate as equal to $CF \cdot A \cdot \frac{{R_{Wo} }}{{R_{Po} }}$ and assumed the same *τ* [8], knowing that in fact the count losses due to pileup are affecting the primary counts to a greater extent than the wide-spectrum counts. The upper limit of the quantifiable range was defined as the activity or R_Wo_ at which the observed count rate decreases (as expected from a paralyzable system), or plateaus (in case a system has absolute maximum count rate limit). Look-up tables were created to retrieve the dead-time correction factor, i.e., the ratio of the true to the observed primary count rates, for a given observed wide-spectrum count rate, R_Wo_, during SPECT acquisition [6, 8].

To study the pileup effect, the repartition of counts between the energy windows was plotted against activity.

### SPECT/CT acquisitions

**Fig. 1:**
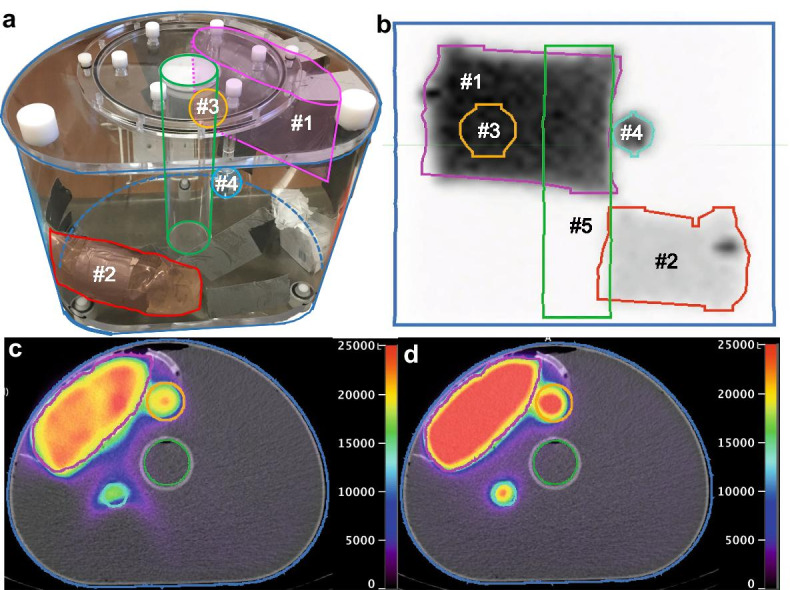
The NEMA phantom (**a**) containing a 500-mL (#1) and a 250-mL (#2) bags, large sphere (#3), small sphere (#4) and the cold-water-filled cylinder (#5). Maximum intensity projection (**b**) and selected transaxial fusion slices from System A with both detectors (**c**; 4.93 GBq total activity) and only detector 1 activated (**d**; 5.12 GBq total activity), respectively. The scale is expressed in terms of counts per pixel

A water-filled NEMA 2012/ IEC 2008 phantom (Biodex Medical Systems, USA) was customized by adding two saline bags of 250 and 500 mL inside the phantom to simulate a kidney and a large liver lesion, respectively, and two spheres to simulate additional lesions (Fig. 1a; Table 1). Acquisitions were performed over up to 26 h: 13 acquisitions of 96 projections (System A) or 9 acquisitions of 90 projections (for each Systems B and C), step-and-shoot mode, non-circular orbit, 128 × 128 matrix, 5 or 10 s per frame depending on the activity. For each dual-detector acquisition, detectors 1 and 2 started at 0° and 180°, respectively. In single-detector mode, the activated detector started at 0° and acquired all projections over 360°. Low-dose CT acquisition was performed subsequently (110 or 120 kVp, 40 or 70 mAs). Data was reconstructed using a vendor-neutral software (SPECTRA Quant, MIM Software, USA) with ordered subset expectation maximization (5 iterations, 6 subsets), CT-based attenuation, resolution recovery, and triple energy window scatter corrections.

**Table 1:** NEMA phantom initial activity distribution

Compartment	Volume (mL)	System A		Systems B and C	
		Activity (GBq)	Activity concentration (MBq/mL)	Activity (GBq)	Activity concentration (MBq/mL)
Large saline bag	500	12.0	24.0	11.0	22.0
Small saline bag	250	1.20	4.81	1.24	4.95
Large sphere	26.5	0.66	24.7	0.54	22.0
Small sphere	11.5	0.31	27.0	0.23	22.0
Cylinder	355	0.0	0.0	0.0	0.0
Remainder of D-shaped compartment	8557	4.12	0.48	3.94	0.46
Whole phantom	9700	18.3	1.89	17.0	1.76

### SPECT quantification and image quality

Using the CT, volumes of interest (VOIs) were manually drawn around the different compartments of the NEMA phantom. The contour of the phantom itself was automatically defined based on the CT (−400 HU threshold). A 200-mL VOI was also drawn in the background of the main compartment, far from the spill out from high-activity objects. The contours of the small saline bag and the whole phantom were both expanded by 1 cm, to include spilled-out counts [6, 14]. The estimated background activity included in the VOI expansion of the small saline bag was subtracted from its total activity. The mean VOI counts per second was quantified, dead-time corrected (by multiplying counts with the dead-time correction factor corresponding to the average acquisition R_Wo_), divided by the actual volume, and compared to the known activity concentration.

## Results

### Planar acquisitions

**Fig. 2:**
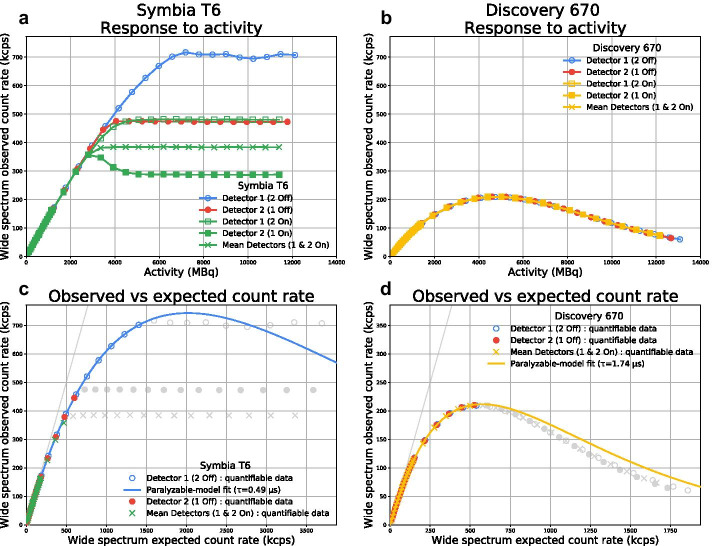
System A (Symbia T6; **a** and **c**) and B (Discovery 670; **b** and **d**) observed count rate vs. activity (**a** and **b**) and expected count rate (**c** and **d**; only quantifiable non-grayed data points fitted to the paralyzable model) during planar acquisitions

As activity increased, the observed count rate of System A depended on detector activation (Fig. 2a). When both activated, detectors 1 and 2 diverged above 375 kcps and eventually plateaued at specific levels (480 and 290 kcps, respectively). However, those same detectors saturated at different levels (700 and 475 kcps, respectively) when they were individually activated. While the count rate drop at higher activity that is typical of a paralyzable system was not as obvious for System A during this particular experiment, we did observe it in the past when acquiring with ^99m^Tc using a more transparent medium-energy collimator (Additional file 1: Fig. S1). Conversely, the response of Systems B and C was independent of the detector configuration and exhibited a maximum observed count rate of 205 kcps, as predicted by Sorenson’s model, before depressing at higher activities (Fig. 2b). For the following results, A1 and A2 refer to System A with only one (detector 1) and both detectors activated, respectively.

### Paralyzable model calibration

System A (CF = 99.97 ± 0.07 cps/MBq; *τ* = 0.4949 ± 0.0004 μs; Fig. 2c) was more sensitive and less prone to dead time than Systems B (CF = 75.37 ± 0.16 cps/MBq; *τ* = 1.738 ± 0.004 μs; Fig. 2d) and C (CF = 87.46 ± 0.30 cps/MBq; *τ* = 1.740 ± 0.008 μs; data not shown because it is similar to Fig. 2d). The different CFs between Systems B and C are only attributable to the collimators’ design. The maximum quantifiable unattenuated ^99m^Tc activities as visually determined from Fig. 2 were equal to 6.58, 2.79, 5.02, and 4.34 GBq, respectively, for Systems A1, A2, B, and C. We confirmed that the systems do not behave as cascaded paralyzable–non-paralyzable systems, as fitting data to such model result in non-paralyzable constant that is smaller than the paralyzable one, which is not allowed (data not shown) [10].

### Pileup effect

**Fig. 3:**
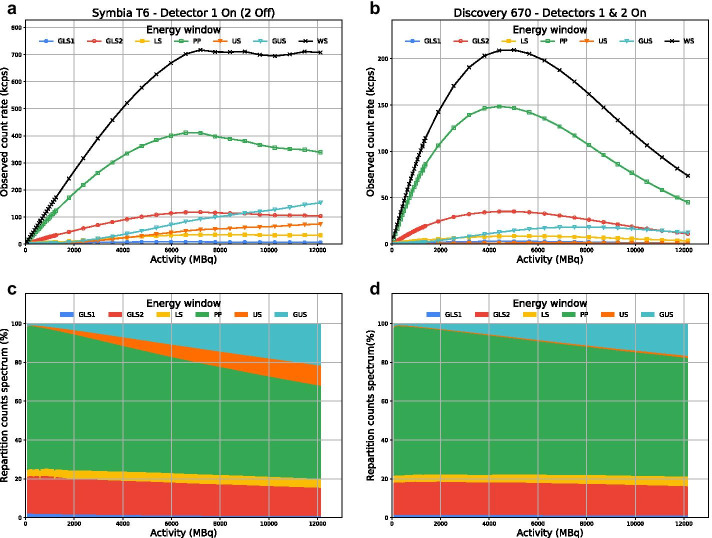
System A1 (Symbia T6 with only detector 1 activated; **a** and **c**) and System B (Discovery 670, both detectors activated; **b** and **d**) observed count rates (**a** and **b**) and percentage of counts (**c** and **d**) for the different energy windows are plotted against activity. GLS1 = general scatter 1 (18–53 keV). GLS2 = general scatter 2 (53–112 keV). LS = lower scatter (112–126 keV). PP = photopeak (126–154 keV). US = upper scatter (154–168 keV). GUS = general upper scatter (168–504 keV). WS = wide spectrum (18–504 keV)

The repartition of counts between the different energy windows during planar acquisitions is presented in Fig. 3 for Systems A1 and B. Because of the pulse pileup, as activity increased, the percentage of counts in the photopeak decreased, while that in both upper scatter windows increased. The phenomenon was more pronounced with System A. Because scatter events are subtracted from photopeak events to obtain the primary counts, the pileup effect amplifies the dead-time loss of primary counts at high count rate. Nevertheless, the Sorenson’s model fits the data well.

### SPECT quantification

**Fig. 4:**
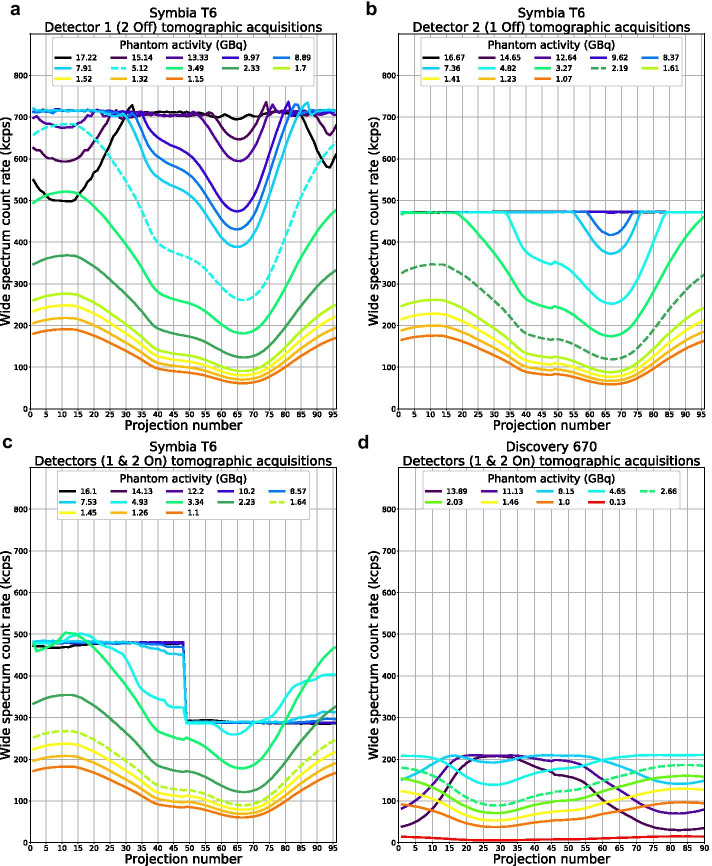
Observed wide-spectrum count rate vs. projection number for System A (Symbia T6) with only the detector 1 (**a**), only the detector 2 (**b**), or both detectors activated (**c**), and System B with both detectors activated (**d**; Discovery 670; visually similar to NM/CT 870 DR) during SPECT acquisitions of the NEMA phantom. The dashed lines correspond to the maximum quantifiable activity

**Fig. 5:**
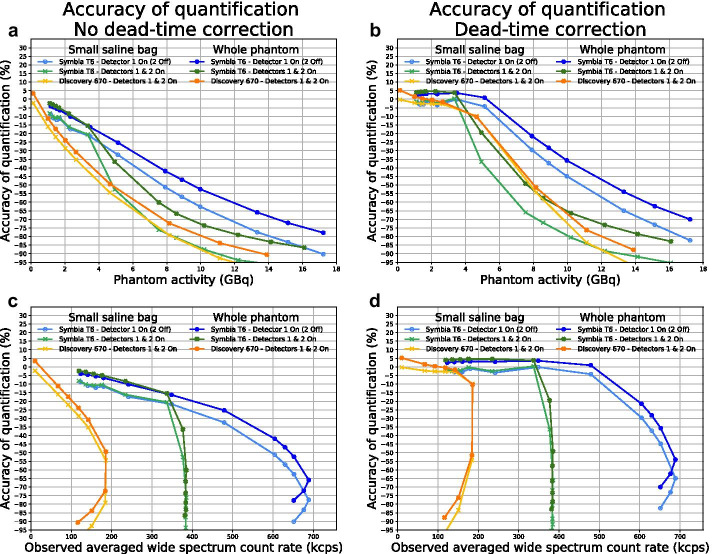
SPECT quantification accuracy for the whole NEMA phantom and the small saline bag (CT-based VOIs expanded by 1 cm for both) *vs.* activity (**a** and **b**) and observed averaged wide-spectrum count rate (**c** and **d**), for System A1 and A2 (Symbia T6 with only detector 1 and both detectors activated, respectively) and System B (Discovery 670 with both detectors activated), without (**a** and **c**) and with (**b** and **d**) dead-time correction using the paralyzable model

The observed count rate per projection during the SPECT acquisitions was consistent with the planar data, showing detector configuration-dependent saturation levels only for System A (Fig. 4). The paralyzable behaviour of systems is again evidenced by lower count rates in angular positions of maximum exposure at high activity (in particular Fig. 4a and d). After dead-time correction, accurate quantification (i.e., less than 5% deviation from true activity) of the heterogeneous attenuating phantom was achieved up to 5.12, 3.34, 2.66, and 2.30 GBq, respectively, for Systems A1, A2, B, and C (Fig. 5). However, it must be noted that for System A2, the phantom placement was favorable to the system design (hottest area primarily imaged by detector 1), and 1.64 GBq would not have exceeded the lower saturation level of detector 2 in any projection (Fig. 4c, dashed line).

### SPECT image quality

With System A2, we observed image artifacts when the activity was greater than 3.34 GBq, attributable to the differential detector behaviours (Fig. 1c). These artifacts were not observed with System A1 (Fig. 1d), B and C.

## Discussion

We previously demonstrated the necessity to correct for dead time in post-therapeutic ^177^Lu imaging with NaI(Tl)-crystal cameras [5]. Here, we comprehensively investigated the divergent detector behaviour of System A at high count rate, which can compromise the accuracy of quantification and dosimetry, as well as the image quality (Fig. 1c) [6]. This issue was suspected in few of our patients treated with high activities (> 20 GBq) of ^177^Lu-octreotate and exhibiting high tumour retention in our personalized peptide receptor radionuclide therapy regimen [2]. In these occasional, extreme clinical situations, we found that the quantitative capacity of System A at high count rate can be extended by performing acquisitions with detector 1 only. When such cases are anticipated, the camera count rate can be verified on the monitor before starting the acquisition in the appropriate dual- or single-detector mode. To avoid an excessive exam duration in single-detector mode, the time per projection can probably be reduced without risking to significantly degrade the image quality owing to the very high count rate.

For practical reasons, we used ^99m^Tc on the basis that, for a given system, the wide-spectrum count rate as the determinant of dead time is expected to be relatively radionuclide-independent. Indeed, for System A, previously characterized as a paralyzable system, we observed a similar wide-spectrum count rate-based *τ* of 0.49 μs for ^99m^Tc, vs. 0.55 μs for ^177^Lu [6], suggesting a limited influence of the distribution of counts within the wide spectrum on the loss of primary counts at high count rate.

To extend the scope of our work, we evaluated two additional SPECT/CT systems (differing only by their collimators) from another leading manufacturer. At 1.74 µs, the *τ* of Systems B and C is substantially larger than that of System A and consistent with that determined by others at 1.66 µs using ^99m^Tc [15]. It is likely that *τ* of Systems B and C would be similar if measured using ^177^Lu. While collimator design can partly account for differences in sensitivity between cameras having otherwise the same crystal thickness, it should not, in principle, affect *τ.*

We later learned that a “fast” acquisition mode is available on Systems B and C (not on System A), which we did not investigate [16]. According to the vendor, this mode enables higher count rates at the expense of a lower spatial resolution and is seldom used for clinical SPECT. While the impact of this fast mode on quantitation and image quality warrants further investigation, we nevertheless compared all systems in their respective “normal” acquisition mode.

As observed by others [17], determining the dead-time constant of a system based only on the photopeak count rate (and adjacent scatter windows) would not take into account the increasing pileup effect (Fig. 3). This would eventually lead to quantification errors. We also previously demonstrated that a dead-time constant based on the photopeak count rate is dependent on the object/subject geometry (i.e., volume and shape of attenuating/scattering medium), while one based on the wide-spectrum count rate is not [6, 8]. Hence, we recommend using the latter to determine the dead time affecting the primary events.

Comparison of dead time from different manufacturers has already been done by others [7]. Our study, however, focused on the response of modern SPECT/CT systems and its impact on quantification. Our results point to some advantages of System A which features higher sensitivity, shorter dead time, and extended QSPECT capacity at very high count rate. However, its maximum observed count rate capacity is limited by an absolute total count rate limit of ~ 700 kcps in both single- or dual-detector mode, rather than the maximum count rate predicted by the Sorenson model of 743 kcps for each detector independently. This is caused by a downstream electronics bottleneck, while the prioritization of the detector 1 in dual-detector mode is the result of the design of this system.

However, new CZT-based SPECT/CT systems with improved energy resolution may become the state-of-the-art technology for personalized theranostic applications in the future [18, 19]. If the downstream electronics would not impose a bottleneck count rate limit (e.g., the plateau seen with System A), a CZT camera equipped with multiple small detector subunits (i.e., each counting only a small fraction of the total events) could in theory exhibit very little dead time at the system level.

## Conclusion

We found significant differences in key features of modern NaI(Tl)-crystal SPECT/CT systems affecting their quantitative performance at high count rate. These are relevant to consider for accurate QSPECT, especially in the context of personalized radiopharmaceutical therapy.

## Supplementary Information

**Additional file 1**: **Fig. S1**. Observed count rate vs. activity with System A (Symbia T6, only detector 1 activated) equipped with medium-energy low-penetration collimators.

## Data Availability

Please contact the corresponding author for the data used in this manuscript.

## Abbreviations

A: Activity
CF: Calibration factor
CV: Coefficient of variation
CZT: Cadmium zinc telluride
QSPECT: Quantitative single-photon emission computed tomography
R_Po_: Observed primary photon count rate
R_Wo_: Observed wide-spectrum photon count rate
τ: Dead-time constant
VOI: Volume of interest
