# Supplementary figures and images for: Quantitative SPECT (QSPECT) at high count rates with contemporary SPECT/CT systems
Source: EJNMMI Phys. 2021 Oct 30;8:73. doi: 10.1186/s40658-021-00421-3 (PMC8557232; doi:10.1186/s40658-021-00421-3)

# Symbia T6 MELP Response to activity

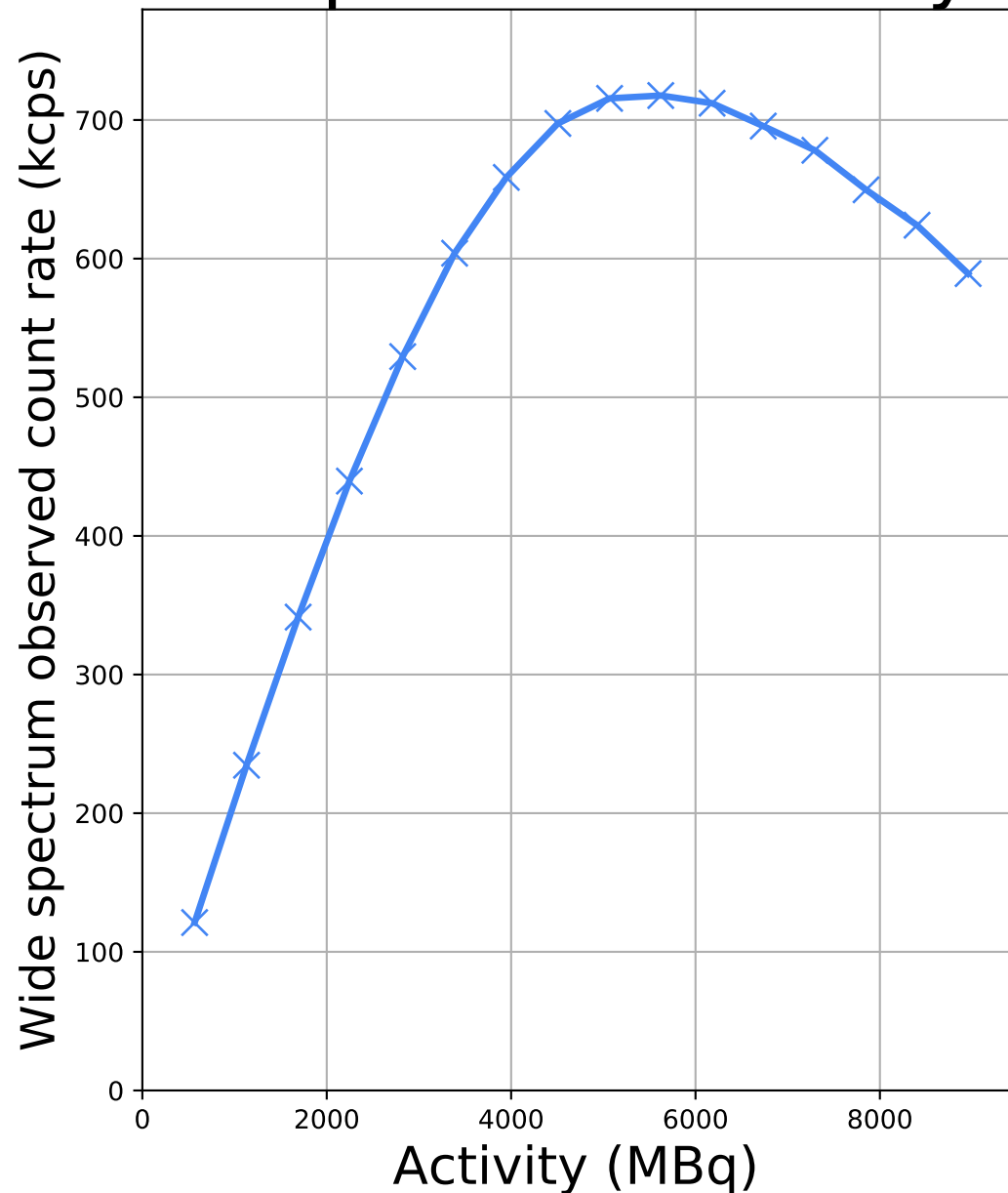

Supplement: Supplementary file 1 — Additional file 1: Fig. S1. Observed count rate vs. activity with System A (Symbia T6, only detector 1 activated) equipped with medium-energy low-penetration collimators. [file 40658_2021_421_MOESM1_ESM.pdf]
